# Awareness of U = U among Sexual and Gender Minorities in Brazil, Mexico, and Peru: Differences According to Self-reported HIV Status

**DOI:** 10.1007/s10461-024-04336-9

**Published:** 2024-04-25

**Authors:** K. A. Konda, J. Qquellon, T. S. Torres, E. H. Vega-Ramirez, O. Elorreaga, C. Guillén-Díaz-Barriga, D. Diaz-Sosa, B. Hoagland, J. V. Guanira, M. Benedetti, C. Pimenta, H. Vermandere, S. Bautista-Arredondo, V. G. Veloso, B. Grinsztejn, C. F. Caceres

**Affiliations:** 1 Keck School of Medicine, University of Southern California, 1845 N Soto St, Los Angeles, CA 90032 USA; 2 Centro de Investigación Interdisciplinaria en Sexualidad, Universidad Peruana Cayetano Heredia, SIDA y Sociedad, Lima, Peru; 3 Instituto Nacional de Infectologia Evandro Chagas, Fundação Oswaldo Cruz (INI-Fiocruz), Rio de Janeiro, Brazil; 4 Instituto Nacional de Psiquiatria Ramon de la Fuente Muñiz, Mexico City, Mexico; 5 Facultad de Estudios Superiores Iztacala, Universidad Nacional Autónoma de México, Mexico City, Mexico; 6 Departmento de Doenças de Condições Crônicas e Infecções Sexualmente Transmissiveis, Brazilian Ministry of Health, Rio de Janeiro, Brazil; 7 Instituto Nacional de Salud Pública (INSP), Mexico City, Mexico

**Keywords:** Latin America, U = U, Sexual and Gender Minorities, HIV

## Abstract

The slogan Undetectable equals Untransmittable (U = U) communicates that people living with HIV (PLHIV) who are on antiretroviral therapy (ART) will not transmit HIV to their sexual partners. We describe awareness of U = U among sexual and gender minorities (SGM) living in Brazil, Mexico, and Peru by self-reported HIV status (PLHIV, negative, unknown) during 2021 using an online survey. We estimated two models using Poisson regression for each population group: Model A including socio-demographic factors (country, gender, age, race, education, and income), and then Model B including taking ART (for PLHIV) or risk behavior, ever-taking PrEP, and HIV risk perception (for HIV-negative or of unknown HIV status). A total of 21,590 respondents were included (Brazil: 61%, Mexico: 30%, Peru: 9%). Among HIV-negative (74%) and unknown status (12%), 13% ever used PrEP. Among PLHIV (13%), 93% reported current use of ART. Awareness of U = U was 89% in both Brazil and Mexico, which was higher than in Peru 64%. Awareness of U = U was higher among PLHIV (96%) than HIV-negative (88%) and HIV-unknown (70%). In multivariate models, PLHIV with lower education were less aware of U = U, while those taking ART were more aware. Among HIV-negative, non-cisgender, lower income, and those with lower education had lower awareness of U = U, while individuals ever using PrEP had higher awareness. In conclusion, awareness of U = U varied by HIV status, socio-demographic characteristics, and HIV risk behavior. The concept of U = U should be disseminated through educational strategies and include a focus on SGM to combat HIV stigma.

## Resumen

Indetectable = Intransmisible (I = I) comunica que las personas que viven con VIH (PVVIH) y reciben tratamiento antirretroviral (TAR) no transmitirán el VIH a sus parejas sexuales. En este estudio, describimos la concienciación sobre I = I entre las minorías sexuales y de género (MSG) de Brasil, México y Perú según el estado de VIH autoreportado (PVVIH, negativo, desconocido) durante 2021 utilizando una encuesta en línea. Se estimaron dos modelos mediante regresión de Poisson para cada grupo: Modelo A, que incluyó factores sociodemográficos (país, sexo, edad, raza, educación e ingresos) y Modelo B, que incluyó recibir TAR (para PVVIH) o comportamiento de riesgo, uso de PrEP y percepción de riesgo (para VIH negativo o desconocido). Se incluyó 21,590 encuestados (Brasil: 61%, México: 30%, Perú: 9%). Entre aquellos negativos para VIH (74%) y con estado desconocido (12%), el 13% utilizó alguna vez PrEP. Entre las PVVIH (13%), el 93% reportó recibir actualmente TAR. La concienciación de I = I fue del 89% tanto en Brasil como en México, superior al 64% de Perú. La concienciación de I = I fue mayor entre PVVIH (96%) que entre los VIH-negativos (88%) y los VIH-desconocidos (70%). En los modelos multivariados, las PVVIH con menor educación eran menos conscientes de I = I, mientras que los que tomaban TAR eran más conscientes. Entre los VIH-negativos, las personas no cisgéneros, con menores ingresos y con menor educación eran menos consciente de I = I, mientras que los que tenían experiencia usando PrEP eran más conscientes. En conclusión, la concienciación sobre I = I varió según el estado serológico de VIH, las características sociodemográficas y el comportamiento de riesgo. El concepto de I = I debe difundirse a través de estrategias educativas, incluyendo un enfoque en MSG para combatir el estigma del VIH.

## Introduction

The slogan ‘Undetectable = Untransmittable’ (U = U) was created to translate the message that people living with HIV (PLHIV) using antiretroviral treatment (ART) with undetectable viral load will not transmit HIV to sex partners [1, 2]. Previous studies supported that the early initiation of ART significantly decreased the transmission of HIV among heterosexual and gay male sero-discordant couples, highlighting the urgent need to prioritize HIV testing and promote treatment as key strategies to stop HIV transmission [3–5].

The Joint United Nations Programme on HIV/AIDS (UNAIDS) and the World Health Organization (WHO) endorse U = U and encourage countries to spread their meaning and knowledge among vulnerable people [6]. In addition, the most recent campaign of the WHO ‘Zero risk’ encourages healthcare providers to promote the message of U = U with easy understanding [7, 8]. Moreover, the unyielding stigma associated with HIV infection has persisted since the beginning of the HIV epidemic. This remains unabated, despite the tremendous success of ART and the conversion of living with HIV to a chronic disease rather than a “death sentence”. U = U is an attempt to lessen the fear associated with HIV infection, reducing stigma and highlighting that PLHIV are able to have high quality of life just like people not living with HIV [9].

The idea behind U = U serves multiple purposes beyond stigma reduction. U = U can allow PLHIV to disclose to their sex partners in a way to avert fear [10]. It can also help to reduce fears about HIV testing as it helps to communicate the success of available HIV treatment [11]. Awareness of U = U has gained traction in some settings. A systematic review found notable variations in levels of U = U awareness among gay, bisexual and other men who have sex with men (MSM), with higher levels in high-income settings such as Europe, Asia, Oceania, and parts of Latin America, while lower levels were reported in African regions [12]. In addition, several studies have reported higher percentages of awareness and acceptability among PLHIV compared to MSM or heterosexuals with seronegative or unknown status [12–15]. However, U = U remains relatively unused and in limited existing reports, is perceived as inaccurate in Latin America. An online study in Brazil reported that 56% of cisgender MSM who self-reported as HIV-negative or unknown status and 20% of PLHIV had concerns about the accuracy of the U = U [16]. In addition, 38% and 26% physicians from Mexico and Brazil, respectively, perceived the U = U slogan as not completely accurate [17]. We did not find studies on the concept of U = U conducted in Peru.

As part of a survey to better understand HIV prevention knowledge among sexual and gender minority populations in Latin America, we collected information on U = U awareness and perception of accuracy. This analysis aims to describe and estimate factors associated with U = U awareness among sexual and gender minorities (SGM) in three Latin American countries by self-reported HIV status.

## Methods

### Study Design

A cross-sectional online survey was conducted between April and August 2021 among SGM aged 18 years or older from Brazil, Peru, and Mexico. Most individuals were recruited via dating apps such as Grindr (19.2%) and Hornet (39.4%), followed by social networks such as Facebook (19.1%), Instagram (17.1%) and WhatsApp (2.4%), and other sources (2.9%). They were asked to provide their informed consent and then to answer a computer-based survey that included information on socio-demographics, self-reported HIV status, experiences with HIV pre-exposure prophylaxis (PrEP) or ART, and risk behaviors, including sexual risk as well as drug and alcohol use. Participants were asked to report their awareness and perceived accuracy of U = U.

### Variables

#### Outcome

Awareness of U = U was assessed with the question, *“Have you heard of the concept of Undetectable = Untransmittable (U = U)?”* with possible answers of ‘Yes’ or ‘No’. Also, we showed the definition of U = U, *“meaning that people living with HIV who maintain an undetectable viral load do not transmit HIV through sex”*, and asked, *“How accurate do you consider the definition of U = U to be?”* with answers categorized as totally accurate, partially accurate, partially inaccurate, totally inaccurate, and I don’t know what “undetectable” means.

### Socio-Demographics

Age was recategorized as 18 to 24, 25 to 34 and ≥ 35 years old. Gender identity was assessed with a question on their self-reported current gender identity and was dichotomized as cisgender men and transgender/non-binary persons. Race or ethnicity was categorized as White, Mixed-race (*Pardo* or *Mestizo*) and Black/Asian/Indigenous. Education level was dichotomized as completed secondary or less and higher than secondary education. We considered the individual monthly income according to each country’s minimum wage in 2021 (Brazil ~ 213 USD/month, Mexico ~ 215 USD/month, and Peru ~ 257 USD/month) and dichotomized into below minimum wage and above minimum wage.

### HIV Status, HIV risk Perception, HIV Incidence Risk Index for MSM

Participants self-reported their HIV status. People living with HIV (PLHIV) answered ‘Yes’ to the question, *“Have you tested positive for HIV?”* while HIV negative individuals answered ‘No’ and reported when they had last tested for HIV (in the last 3 months, in the last 6 months, in the last year, or over a year ago). Participants were classified as having unknown HIV status if they answered that they had never been tested for HIV or did not want to answer whether they had tested positive for HIV. HIV risk perception was measured with the question, *“Considering your current sexual practices, in your opinion, what is your risk of getting HIV in the next year?”* which was dichotomized as low/moderate (none, low, and some risk) and high (high risk/certainty of infection). The HIV Incidence Risk Index for MSM (HIRI-MSM) to assess the risk of HIV infection was also used [18]. The index was built including 6 questions with scored answers about age, number of sexual partners, number of HIV positive sexual partners, number of condomless receptive anal sex acts, number of condomless insertive anal sex acts with PLHIV, and the use of stimulants such as cocaine, crack, ecstasy, and gamma-hydroxybutyrate in the last 6 months. Scores of 10 or higher were considered a “high risk” [18, 19]. The HIRI index has been used previously among SGM from Latin America [20–22].

### Adherence to Antiretroviral Treatment

Among participants who self-reported as PLHIV, we measured if they are currently using ART with the question, *“Are you taking antiretroviral treatment?”*, with ‘Yes’ or ‘No’ answer choices.

### Experience with HIV Pre-exposure Prophylaxis (PrEP)

Among participants who self-reported as HIV negative or of unknown HIV status, prior or current experience with PrEP was evaluated using the following questions: *“Have you ever taken PrEP in your life?”* and, *“Are you currently taking PrEP?”* Responses to these questions were then categorized into ‘Ever taking PrEP’ and ‘Never taking PrEP’. Also, we described the awareness of PrEP with the question: *“Have you heard of Pre-exposure Prophylaxis (PrEP)?”*, with possible answers of ‘Yes’ or ‘No’.

### Statistical Analysis

We described frequencies of socio-demographics, HIV status, use of PrEP, and risk behavior characteristics per country (Brazil, Mexico, and Peru) and used chi-square tests to compare them by self-reported HIV status (PLHIV, negative and unknown). In the exploratory analysis, we used Poisson regression to calculate prevalence ratios (PR) of factors associated with awareness of U = U stratified by self-reported HIV status. For each group PLHIV, negative and unknown, we estimated two adjusted models, the model A adjusted only for socio-demographic factors: country, gender, age, race, education, and income. While model B adjusted for additional relevant variables depending on the population type. Among individuals who self-reported as HIV negative or of unknown HIV status, model B also adjusted for ever taking PrEP, HIV risk perception, and HIRI-MSM index. While among individuals who self-reported as living with HIV, model B was also adjusted for adherence to ART.

## Results

**Fig. 1:**
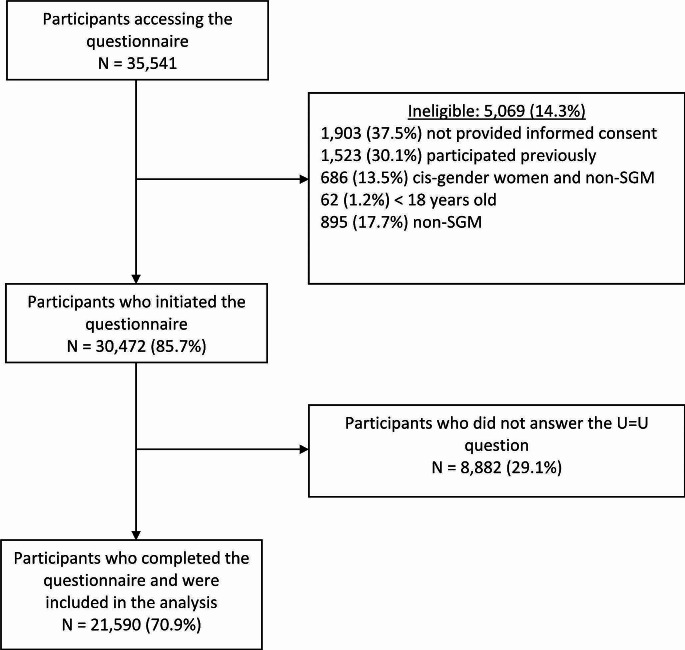
Study flow-chart

Among 35,541 individuals who accessed the questionnaire, 5069 (14.3%) were ineligible for reasons including not providing informed consent (37.5%), participating previously (30.0%), cisgender women who self-identified as heterosexual or lesbian (13.5%), aged < 18 years old (1.2%), and not identifying as a sexual or gender minority (cisgender men who self-identified as heterosexual) (17.7%) (see Fig. 1). Of the eligible individuals (*N* = 30,472), 21,590 (70.9%) completed the questionnaire and were included in the analysis. The mean age was 33.5 (standard deviation [SD] ± 9.4) years, 95.4% identified as cisgender men, 0.6% as transgender women, 0.4% as transgender men, 0.9% as queer, 1.5% as non-binary, 0.1% as cisgender women and 1.1% as others and most participants (66.7%) had completed more than secondary education. A total of 13.3% of participants reported living with HIV, 74.4% reported being HIV negative and 12.3% reported not knowing their HIV status. Among PLHIV, 93.3% were receiving ART. Among HIV-negative and of unknown HIV status individuals, 13.2% ever used PrEP, 87.2% were aware of PrEP, 9.4% self-perceived themselves as at high risk for HIV, and 50.6% were classified as high-risk according to the HIRI index. Overall, awareness of U = U was 86.6% with higher awareness in Mexico (89.2%) and Brazil (88.6%) as compared to Peru (63.7%). Awareness was higher among PLHIV (95.9%) than individuals self-reporting as HIV negative (87.7%) or having an unknown HIV status (69.9%).

Participants characteristics according to HIV status differ by country (see Table 1). We found a significant difference in U = U awareness between each HIV status group in the three countries (all with a *p* < 0.001). Among PLHIV, awareness was higher in Mexico (98.9%) and in Brazil (95.7%) as compared to Peru (85.3%), among HIV negative, awareness was higher in Mexico (90.2%) and in Brazil (89.1%) as compared to Peru (66.1%), and among unknown HIV status, awareness was higher in Brazil (75.2%) and in Mexico (74.3%) as compared to Peru (43.2%). Regarding PrEP, more HIV negative than unknown HIV status participants had previous experience with it in the three countries (all with a *p* < 0.001), with a higher proportion in Brazil (18.7%). According to the risk score by HIRI-MSM index, there were differences in risk score between HIV negative and unknown status in the three countries; however, a significant difference in HIV risk perception was found only in Mexico. Considering socio-demographic characteristics, there were more PLHIV, HIV negative, and unknown HIV status Peruvians identified as non-cisgender (15.5%, 9.2%, and 13.4%, respectively), young people aged 18–24 (30.2%, 39.4% and 64.1%, respectively) and individuals receiving the minimum wage or less per month (56.5%, 44.7% and 63.8%, respectively) than in Mexico and Brazil.

**Table 1:** Characteristics of an online sample of sexual and gender minorities from Brazil, Mexico, and Peru according to HIV sexual

			Brazil				Mexico				Peru				
	Characteristics	Total *N* = 21,590 (100.0%)	PLHIV 1589 (12.1%)	HIV Negative 10,293 (78.4%)	Unknown HIV Status 1252 (9.5%)	*p*value^a^	PLHIV 1042 (15.8%)	HIV Negative 4555 (69.2%)	Unknown HIV Status 987 (15.0%)	*p* **value** ^a^	PLHIV 252 (13.5%)	HIV Negative 1208 (64.5%)	Unknown HIV Status 412 (22.0%)	*p* **value** ^a^	
**Gender Identity** **(*n*** **= 20,589)**						0.069				**0.002**				**0.003**	
	Cisgender men	20,604 (95.4)	1554 (97.8)	10,076 (97.9)	1213 (96.9)		979 (93.9)	4227 (92.8)	888 (90.0)		213 (84.5)	1097 (90.8)	357 (86.6)		
	Non-cisgender	985 (4.6)	35 (2.2)	216 (2.1)	39 (3.1)		63 (6.1)	328 (7.2)	99 (10.0)		39 (15.5)	111 (9.2)	55 (13.4)		
**Age (years)**						**< 0.001**				**< 0.001**				**< 0.001**	
	18–24	3649 (16.9)	103 (6.5)	1158 (11.3)	310 (24.8)		93 (8.9)	816 (17.9)	353 (35.8)		76 (30.2)	476 (39.4)	264 (64.1)		
	25–34	9243 (42.8)	561 (35.3)	4478 (43.5)	508 (40.5)		480 (46.1)	2137 (46.9)	369 (37.4)		103 (40.9)	519 (43.0)	88 (21.4)		
	≥ 35	8698 (40.3)	925 (58.2)	4657 (45.2)	434 (34.7)		469 (45.0)	1602 (35.2)	265 (26.8)		73 (29.0)	213 (17.6)	60 (14.5)		
**Race (***n* **= 20,688)**						**< 0.001**				**0.001**				0.078	
	White	8612 (41.6)	864 (54.4)	5854 (56.9)	631 (50.4)		127 (14.2)	737 (18.5)	163 (20.1)		32 (12.7)	141 (11.7)	63 (15.3)		
	Mixed	9552 (46.2)	482 (30.3)	3083 (29.9)	412 (32.9)		695 (77.6)	2987 (75.1)	577 (71.2)		171 (67.9)	875 (72.4)	270 (65.5)		
	Black/Asian/Indigenous	2524 (12.2)	243 (15.3)	1356 (13.2)	209 (16.7)		73 (8.2)	253 (6.4)	70 (8.7)		49 (19.4)	192 (15.9)	79 (19.2)		
**Education level**						**< 0.001**				**< 0.001**				**< 0.001**	
	≤ Secondary education	7196 (33.3)	649 (40.8)	3265 (31.7)	635 (50.7)		303 (29.1)	1233 (27.1)	449 (45.5)		85 (33.7)	364 (30.1)	213 (51.7)		
	> Secondary education	14,394 (66.7)	940 (59.2)	7028 (68.3)	617 (49.3)		739 (70.9)	3322 (72.9)	538 (54.5)		167 (66.3)	844 (69.9)	199 (48.3)		
**Monthly income** ^b^ **(*n*** **= 21,022)**						**< 0.001**				**< 0.001**				**< 0.001**	
	≤ Minimum wage	4940 (23.5)	350 (22.0)	2050 (19.9)	419 (33.5)		168 (16.9)	794 (18.5)	319 (34.4)		131 (56.5)	485 (44.7)	224 (63.8)		
	> Minimum wage	16,082 (76.5)	1239 (78.0)	8243 (80.1)	833 (66.5)		823 (83.1)	3505 (81.5)	608 (65.6)		101 (43.5)	600 (55.3)	127 (36.2)		
**U = U awareness**						**< 0.001**				**< 0.001**				**< 0.001**	
	No	2892 (13.4)	69 (4.3)	1121 (10.9)	310 (24.8)		11 (1.1)	447 (9.8)	254 (25.7)		37 (14.7)	409 (33.9)	234 (56.8)		
	Yes	18,698 (86.6)	1520 (95.7)	9172 (89.1)	942 (75.2)		1031 (98.9)	4108 (90.2)	733 (74.3)		215 (85.3)	799 (66.1)	178 (43.2)		
**Prior experience with PrEP** **(*n*** **= 16,743)**						**< 0.001**				**< 0.001**				**< 0.001**	
	No	14,538 (86.8)	N/A	7382 (81.3)	796 (97.2)		N/A	4029 (92.1)	873 (97.6)		N/A	1089 (90.7)	369 (97.4)		
	Yes	2205 (13.2)	N/A	1697 (18.7)	23 (2.8)		N/A	343 (7.9)	21 (2.4)		N/A	111 (9.3)	10 (2.6)		
**HIV risk perception** ^c^ **(*n*** **= 17,524)**						0.101				**0.007**				0.067	
	None/Low/moderate	15,870 (90.6)	N/A	8921 (90.6)	950 (92.1)		N/A	4102 (90.9)	885 (93.7)		N/A	798 (84.1)	214 (88.8)		
	High	1654 (9.4)	N/A	927 (9.4)	81 (7.9)		N/A	408 (9.1)	60 (6.3)		N/A	151 (15.9)	27 (11.2)		
**HIRI-MSM (*n*** **= 18,707)**^d^						**< 0.001**				**< 0.001**				**< 0.001**	
	Low risk	7912 (42.3)	N/A	3987 (38.7)	675 (53.9)		N/A	1865 (40.9)	500 (50.7)		N/A	610 (50.5)	275 (66.7)		
	High risk	10,795 (57.7)	N/A	6306 (61.3)	577 (46.1)		N/A	2690 (59.1)	487 (49.3)		N/A	598 (49.5)	137 (33.3)		

*PLHIV* people living with HIV, *PrEP* pre-exposure prophylaxis, *N/A* not applicable

^a^ Chi-square test

^b^ According to each country’s monthly minimum wage in 2021: Brazil ~ 213 USD/month, Mexico ~ 215 USD/month and Peru ~ 257 USD/month

^c^ In the coming year

^d^ The HIV incidence risk index for men who sex with men (HIRI-MSM) scale was built including six questions about age, sexual partners, sex partners living with HIV, condomless receptive anal sex, condomless insertive anal sex and use of drugs or stimulants. Scores ≥ 10 = “high risk”, scores < 10 = “low risk”

**Fig. 2:**
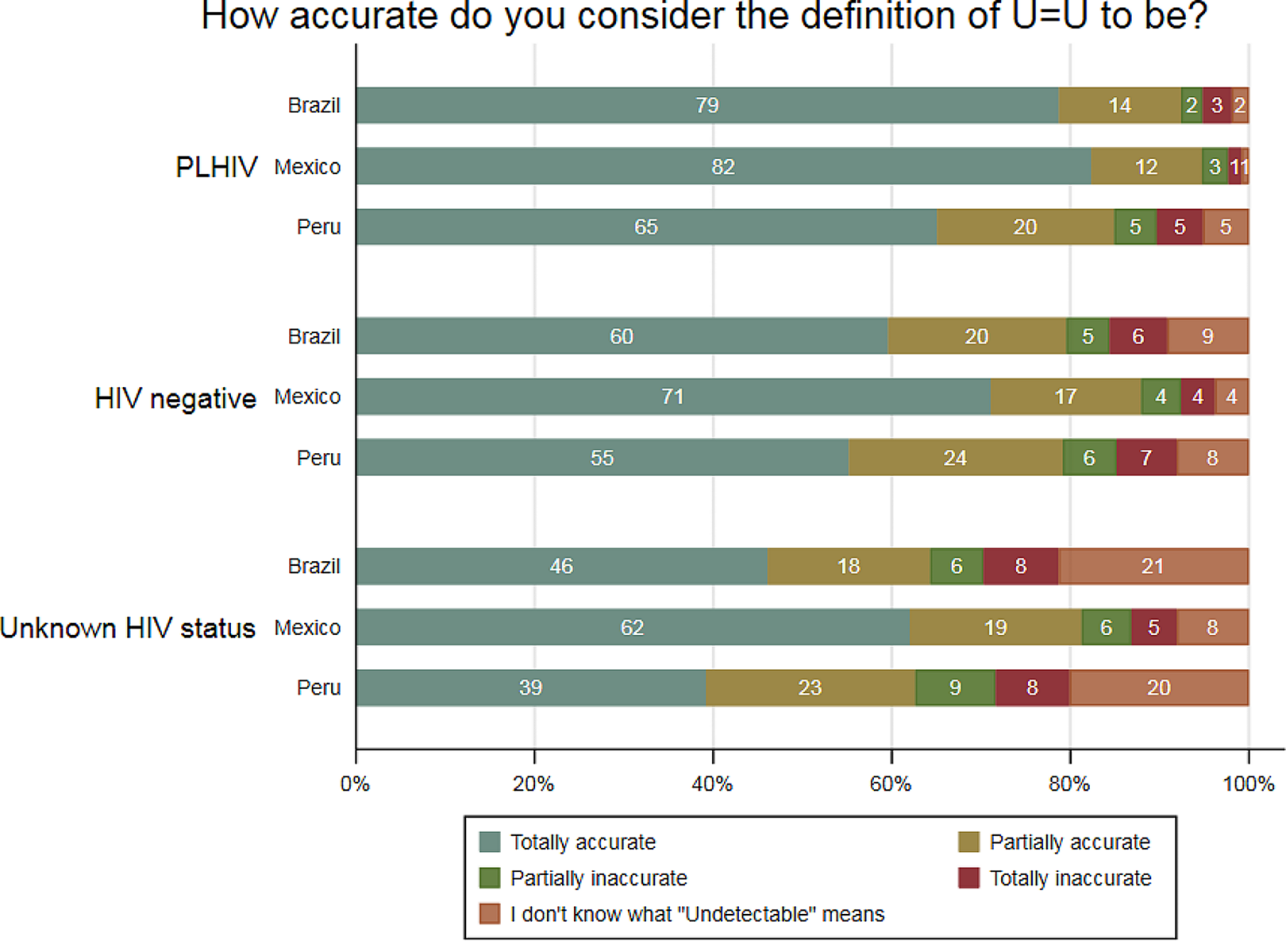
Percentages of perceived accuracy of U = U according to self-reported HIV status

The overall perception of U = U as totally accurate was 63.3%; however, it was higher in PLHIV (78.8%) compared to HIV negative (62.5%) and unknown status (51.1%). Also, the perceived accuracy varied by country (Fig. 2). Among PLHIV, most perceived U = U as totally accurate and this was higher in Mexico (82.3%) and Brazil (78.7%), compared to Peru (65.1%). Among HIV negative, most perceived as totally accurate was higher in Mexico (71.1%) compared to Brazil (59.6%) and Peru (55.2%). Also, among individuals who did not know their HIV status, the percentage who perceived U = U as totally accurate was higher in Mexico (62.0%) compared to Brazil (46.1%) and Peru (39.2%). Importantly, among those with unknown HIV status, 21.3% of Brazilian and 20.1% of Peruvians did not know what “undetectable” means, while this was only 8% of Mexicans.

Regarding the adjusted multivariate models (see Table 2), the results from Model A showed socio-demographic characteristics associated with each HIV status group. Among PLHIV participants, having complete secondary education or less was significantly associated with decreased U = U awareness (aPR: 0.96 [CI: 0.95–0.98]). Among those who self-reported being HIV negative, U = U awareness was less frequent among non-cisgender male participants, those aged 35 years or older, those who self-reported mixed race, those with completed secondary education or less, and those earning one minimum wage or less per month (aPR: 0.91 [CI: 0.87–0.95], aPR: 0.95 [CI: 0.93–0.97], aPR: 0.98 [CI: 0.97–1.00], aPR: 0.93 [CI: 0.92–0.94], and aPR: 0.95 [CI: 0.93–0.96], respectively). Among the unknown HIV status group, being non-cisgender, aged 35 years or older and receiving one minimum wage or less per month were negatively associated with U = U awareness (aPR: 0.87 [CI: 0.76–0.99], aPR: 0.90 [CI: 0.83–0.97] and aPR: 0.91 [CI: 0.85–0.97], respectively).

**Table 2:** Characteristics Associated with U = U Awareness among an online sample of Sexual and Gender Minorities from Brazil, Mexico, and Peru according to HIV status

Characteristics		PLHIV		HIV Negative		Unknown HIV Status	
		Model A aPR [95% IC]	Model B aPR [95% IC]	Model A aPR [95% IC]	Model B aPR [95% IC]	Model A aPR [95% IC]	Model B aPR [95% IC]
**Country**							
	Brazil	Ref.	Ref.	Ref.	Ref.	Ref.	Ref.
	Mexico	**1.03 [1.02–1.05]**	**1.03 [1.02–1.05]**	**1.02 [1.01–1.04]**	1.00 [0.99–1.02]	1.01 [0.95–1.06]	0.94 [0.89-1.00]
	Peru	**0.91 [0.87–0.96]**	**0.91 [0.86–0.96]**	**0.76 [0.73–0.79]**	**0.78 [0.74–0.81]**	**0.60 [0.53–0.68]**	**0.60 [0.52–0.70]**
**Gender identity**							
	Cisgender men	Ref.	Ref.	Ref.	Ref.	Ref.	Ref.
	Non-cisgender	0.97 [0.91–1.02]	0.98 [0.93–1.03]	**0.91 [0.87–0.95]**	**0.95 [0.92–0.99]**	**0.87 [0.76–0.99]**	0.88 [0.76–1.01]
**Age (years)**							
	18–24	Ref.	Ref.	Ref.	Ref.	Ref.	Ref.
	25–34	0.99 [0.96–1.03]	0.98 [0.95–1.02]	0.99 [0.97–1.01]	1.00 [0.98–1.02]	1.01 [0.95–1.08]	0.99 [0.92–1.06]
	≥ 35	0.99 [0.95–1.03]	0.98 [0.94–1.01]	**0.95 [0.93–0.97]**	**0.97 [0.95-1.00]**	**0.90 [0.83–0.97]**	**0.87 [0.81–0.95]**
**Race**							
	White	Ref.	Ref.	Ref.	Ref.	Ref.	Ref.
	Mixed	0.98 [0.97-1.00]	0.99 [0.97–1.01]	**0.98 [0.97-1.00]**	0.99 [0.98-1.00]	0.96 [0.91–1.02]	0.98 [0.92–1.03]
	Black/Asian/Indigenous	0.97 [0.94-1.00]	0.98 [0.96–1.01]	0.98 [0.96-1.00]	0.98 [0.96-1.00]	0.95 [0.87–1.03]	0.93 [0.85–1.02]
**Education level**							
	≤ secondary education	**0.96 [0.95–0.98]**	**0.97 [0.95–0.99]**	**0.93 [0.92–0.94]**	**0.95 [0.93–0.96]**	0.96 [0.91–1.02]	0.95 [0.89–1.01]
	> secondary education	Ref.	Ref.	Ref.	Ref.	Ref.	Ref.
**Monthly income**							
	≤ minimum wage	0.98 [0.96-1.00]	0.99 [0.96–1.01]	**0.95 [0.93–0.96]**	**0.96 [0.94–0.97]**	**0.91 [0.85–0.97]**	0.94 [0.88-1.00]
	> minimum wage	Ref.	Ref.	Ref.	Ref.	Ref.	Ref.
**Prior experience with PrEP**							
	No	N/A	N/A	-	Ref.	-	Ref.
	Yes				**1.07 [1.06–1.08]**		**1.13 [1.01–1.28]**
**HIV risk perception**							
	Low/moderate	N/A	N/A	-	Ref.	-	Ref.
	High				1.00 [0.98–1.02]		1.01 [0.92–1.12]
**HIRI-MSM**							
	Low risk	N/A	N/A	-	Ref.	-	Ref.
	High risk				1.01 [1.00-1.02]		1.02 [0.96–1.07]
**Adherence to ART**							
	No	-	Ref.	N/A	N/A	N/A	N/A
	Yes		**1.18 [1.10–1.27]**				

*aPR* adjusted prevalence ratio. Model A was adjusted for socio-demographic variables such as country, gender, age, race, education and monthly income. Model B was adjusted for variables included in the model A and ART adherence (for PLHIV) or prior experience with PrEP, HIV risk perception and HIRI-MSM (for HIV-negative or unknown HIV status). *PLHIV* people living with HIV, *PrEP* pre-exposure prophylaxis, *HIRI-MSM* HIV incidence risk index for men who sex with men. *ART* Antiretroviral treatment, *N/A* not applicable

In the second multivariate model, awareness of U = U among PLHIV was positively associated with adherence to ART (aPR: 1.18 [CI: 1.10–1.27]), and negatively associated with having a complete secondary education or less (PR: 0.97 [CI: 0.95–0.99]). Among the HIV-negative group, ever taking PrEP increased awareness of U = U (aPR: 1.07 [CI: 1.06–1.08]). For this group, the associations with the sociodemographic variables of the first model were maintained, except for self-reported race. Finally, among the Unknown HIV status group, ever taking PrEP was associated with increased awareness of U = U (aPR: 1.13 [CI: 1.01–1.28]), whereas aged 35 years or older decreased awareness of U = U (aPR: 0.87 [CI: 0.81–0.95]).

## Discussion

This study describes the awareness of U = U among SGM from Brazil, Mexico, and Peru, and investigates differences according to self-reported HIV status. Awareness of U = U among SGM in Latin America was high but varied by HIV status and country. PLHIV were more aware of U = U than HIV-negative and unknown HIV status individuals. However, we showed that Peruvians were less aware of U = U than Mexicans and Brazilians regardless of HIV status. PLHIV who reported adherence to ART, and HIV-negative/unknown status who ever used PrEP were more likely to be aware of U = U. PLHIV/HIV-negative who reported having lower education (completed secondary education or less), HIV-negative self-identified as non-cisgender and receiving lower income (one minimum wage or less per month), and HIV negative/unknown status of older age (35 + years) were less likely to be aware of U = U. Campaigns to promote and explain U = U should be tailored for individuals with these characteristics.

Our study found a higher U = U awareness (86.6%) compared to the Latin America MSM Internet Survey (LAMIS) (52.0%) [23]. In contrast to our study, individuals were recruited from 18 Latin American countries, most of them, via dating apps (76%). In addition, we found that Brazil and Mexico showed the highest awareness compared to Peru. Similarly, the LAMIS study reported that awareness of U = U was higher in Brazil (64%) compared to other Latin American countries (which varied between 42 and 49%) [23]. Also, Peru has previously reported lower proportions of PrEP awareness and willingness to use PrEP among MSM compared to Mexico and Brazil [24]. These findings could be related to the stigma of healthcare settings, which is negatively associated with HIV prevention and treatment [25]. UNAIDS reported that 20.7% of PLHIV in Peru experienced discrimination in healthcare settings, additionally, the progress toward the 95-95-95 target was lower in Peru (86%) compared to Brazil (91%) [26]. In addition, we found that PLHIV (95.9%) had a higher awareness of U = U than HIV-negative (87.7%) or unknown-status (69.9%) individuals. Importantly, U = U awareness among each HIV status group varied according to the country. Previous research has confirmed higher rates of U = U awareness among PLHIV from SGM populations (between 74.0 and 95.8%) than HIV-negatives/unknown status (between 46.8 and 85.5%) [13, 27–30].

The perceived accuracy also varied according to the HIV group status and the country. Among PLHIV, this study found higher frequencies of Mexicans (94.7%) and Brazilians (92.4%) who perceived U = U as accurate (totally or partially) compared to Peruvians (84.9%). Previous research has reported the following percentages of U = U accuracy among the different PLHIV populations: up to 90.1% in Brazil [16, 31], 83.9% in the USA [14], 80.4% in Italy [29], and 70.5% in Australia [32]. One of the reasons for finding higher frequencies than other studies may be that we only included individuals who self-identified as part of SGM populations. Moreover, a previous study among HIV physicians found that a majority of Mexicans (62.0%) reported having received training on U = U, while more Brazilians (74.0%) perceived the U = U slogan as completely accurate [17]. No similar studies were found in Peru. This evidence suggests a greater potential for transmitting the U = U message in clinical settings, thereby enhancing its acceptability. Additionally, campaigns have been promoted in Brazil to disseminate the U = U slogan targeting PLHIV and MSM [16]. However, among HIV-negatives and unknown HIV status who perceived U = U as totally or partially accurate, Brazil and Peru reported similar frequencies and considerably lower than those reported by Mexico. Compared to previous studies [13, 14, 16, 29, 31, 33], we found higher proportions, even in Peru. As Rendina et al. explained, this could demonstrate that acceptance of the U = U message has been increasing over time [14]. Interestingly, among individuals of HIV unknown status, high proportions of participants who did not know what “undetectable” meant were found in Brazil (21.3%) and Peru (20.1%). These percentages were similar to Rendina et al. findings in their 2018 survey [14], and even almost double Torres et al. findings in HIV-negative/unknown cisgender MSM (10.8%) and HIV-negative/unknown other populations (9.0%) [16].

Among PLHIV, having completed secondary education or less was associated with lower U = U awareness, while being adherent to ART was associated with higher awareness. These findings were consistent with Huntingdon et al. who reported that PLHIV in Australia identified with a minority cultural background, with lower educational attainment, and recruited using community sources rather than clinical sources had less confidence in U = U [32]. Being aware of U = U has been related to promoting ART initiation and adherence, including improving health indicators such as self-reported viral suppression, optimal physical health, optimal mental health, feeling comfortable sharing their HIV status, and discussing private issues and concerns about HIV transmission with a healthcare provider [27]. Herein ever use of PrEP may be another indicator of increased contact with updated information about HIV, as shown with the association found with knowledge of U = U.

Among HIV-negative individuals, self-identifying as non-cisgender, being older than 35 years, earning minimum wage or less, and having a lower level of education decreased U = U knowledge. A study showed that social position can influence the effectiveness of the U = U message, a higher score after counting marginalized social characteristics of the gay community (trans, a person of color, Indigenous, born abroad, bisexual or straight, not out, struggling with money, not college educated, and not participating in LGBTQ2S + activism/groups) decreased the knowledge of U = U [28]. Concerning age, previous studies have reported that there was better awareness of U = U at older ages, which contradicts our finding [29, 34]. It is important to recognize that although Peru had the highest proportion of individuals unaware of U = U, it was not the country with the highest frequency of lower education levels. This suggests that messages around U = U have been through other sources.

The association of U = U awareness with ever use of PrEP among HIV-negative and unknown HIV status individuals could indicate that PrEP acts as a vehicle for increased understanding of HIV prevention and care overall. Benefits of receiving PrEP services, aside from decreasing the risk of acquiring HIV, include the opportunity to increase knowledge about HIV prevention, understand the message of “Treatment as Prevention”, accurately recognize the meaning of “undetectable”, increase awareness and confidence in U = U, and decrease perceived stigma in health care [35–37]. Between 2018 and 2021, the largest PrEP implementation study in Latin America demonstrated the feasibility of same-day oral PrEP use among MSM and transgender women in Brazil, Mexico, and Peru [38, 39]. Suggesting greater outreach of SGM populations to health services and campaigns focused on HIV prevention.

We acknowledge that this study does have limitations. Our study was based on an online survey, with no probability sampling, we cannot say that this is a representative sample. All individuals participating had to have internet access. The cross-sectional design can only show associations. Although a brief explanation of U = U was provided, we only have brief information on the participants’ views of this concept. As this is an online survey, the HIV status of respondents is based on their self-report, individuals may not answer truthfully (social desirability and recall biases).

## Conclusions

Broad educational strategies, including teaching about the concept of U = U with a focus on SGM vulnerable to HIV infection, are urgent to decrease stigma against PLHIV. Programs such as PrEP and HIV testing can increase awareness through routine counseling associated with these prevention programs.
